# Predicting the pattern and severity of chronic post-stroke language deficits from functionally-partitioned structural lesions

**DOI:** 10.1016/j.nicl.2018.03.011

**Published:** 2018-03-16

**Authors:** Ajay D. Halai, Anna M. Woollams, Matthew A. Lambon Ralph

**Affiliations:** Neuroscience and Aphasia Research Unit, Division of Neuroscience & Experimental Psychology, Faculty of Biology, Medicine and Health, University of Manchester, UK

**Keywords:** Aphasia, Prediction, Principal component analysis, Regression, Stroke

## Abstract

There is an ever-increasing wealth of knowledge arising from basic cognitive and clinical neuroscience on how speech and language capabilities are organised in the brain. It is, therefore, timely to use this accumulated knowledge and expertise to address critical research challenges, including the ability to predict the pattern and level of language deficits found in aphasic patients (a third of all stroke cases). Previous studies have mainly focused on discriminating between broad aphasia dichotomies from purely anatomically-defined lesion information. In the current study, we developed and assessed a novel approach in which core language areas were mapped using principal component analysis in combination with correlational lesion mapping and the resultant ‘*functionally*-*partitioned*’ lesion maps were used to predict a battery of 21 individual test scores as well as aphasia subtype for 70 patients with chronic post-stroke aphasia. Specifically, we used lesion information to predict behavioural scores in regression models (cross-validated using 5-folds). The winning model was identified through the adjusted R^2^ (model fit to data) and performance in predicting holdout folds (generalisation to new cases). We also used logistic regression to predict fluent/non-fluent status and aphasia subtype. Functionally-partitioned models generally outperformed other models at predicting individual tests, fluency status and aphasia subtype.

## Highlights

• Predict the pattern and level of language deficits found in chronic aphasic patients
• Use principal component analysis to identify functional lesion maps
• Functionally-partitioned lesion maps used as predictor variables instead of lesion volume
• Functionally-partitioned lesion model plus age produced the best regression model
• Model can successfully predict fluent/non-fluent types and aphasia classification

## 1 Introduction

Left hemisphere stroke often results in disrupted speech and language processes (aphasia). Under the single umbrella term of ‘aphasia’ there are considerable variations in patients' language and cognitive presentation, in both the pattern and severity of impairment to different language activities (e.g., comprehension, naming, reading, writing, speech, etc.). The consequence of this significant diversity is that individual patients will need very different types of intervention and clinical management (e.g., patients with primary comprehension or phonological deficits). By utilising fMRI in healthy participants (Price, 2010, Price, 2012) and voxel-lesion symptom mapping (Bates et al., 2003) in aphasic patients, cognitive and clinical neuroscience has made considerable strides in mapping language performance and the underpinning cognitive mechanisms to different brain regions. Despite being a crucial step for clinical application, the reverse mapping – using neuroimaging results to predict individual aphasic profiles or types – has only been attempted by a limited number of studies. The key aim of this investigation, therefore, was to embark on using new methods to generate lesion-based models which are able to predict both the detailed language profile of individual patients as well as their aphasia classification. For clarity, in this study we use prediction-based inference to determine how neural data can predict the current behavioural status using a k-fold cross validation approach. Future studies will be able to test whether similar models can offer accurate prediction in the temporal sense (using neural data to predict future behaviour). Indeed, the chronic stroke lesion is apparent long before patients' long-term language and cognitive abilities have stabilised (the partial, gradual recovery that most patients demonstrate extends to at least nine to twelve months post onset). Accordingly, accurate lesion-based prediction models would have considerable clinical utility, including improvements in the type of information that can be offered to patients and carers, enhanced clinical management planning, and appropriate patient stratification to treatment plans.

Studies using neural lesion information to predict behavioural outcomes have yielded inconsistent results. For example, earlier studies reported little advantage of using lesion information in improving predictions (Hand et al., 2006; Johnston et al., 2002; Johnston et al., 2007; Lazar et al., 2008; Willmes and Poeck, 1993). In contrast, more recent studies have found that models, designed to predict a single feature of aphasic performance or aphasia type, can be improved by including lesion information (Hope et al., 2013; Saur et al., 2010; Schiemanck et al., 2006; Thijs et al., 2000; Yourganov et al., 2015). For example, Hope et al. (2013) developed a predictive model using basic demographic information (age, gender, etc.) and structural lesion information obtained from a high-resolution T1-weighted image (lesion size and atlas-based lesions) to predict a composite speech production score (and its constituent individual speech test results), with the winning model containing time post-onset, lesion volume and 35 atlas-based predictors. They showed that the model could predict patients' composite speech production score over the first 200 months post-stroke. In addition, the same group used anatomical regions to predict 22 subtests scores of the Comprehensive Aphasia Test (Swinburn et al., 2005) for mono-/bi-lingual patients (Hope et al., 2015). Another study used ridge regression in order to predict behavioural scores across seven domains (left/right motor, language, attention bias, verbal memory and spatial memory) in acute stroke cases (<2 weeks) (Corbetta et al., 2015) - though, language was identified in a broad sense and thus the study did not allow for predictions of specific language deficits. Other groups have used support vector machines (SVM) trained on atlas-based lesion parcellations to predict six out of ten pairwise binary contrasts between aphasia subtypes at above chance levels (Yourganov et al., 2015). Saur et al. (2010) also used SVMs in order to predict patients' chronic outcome status (a binary classification; good/bad) as well as the type of improvement from the acute to chronic stage (good/bad). They found that age and a composite language recovery score (LRS) achieved above chance classification (62%). It is important to note that this particular study made use of fMRI measurements and showed that the fMRI data within targeted language areas improved prediction accuracy improved significantly (~86%), suggesting that functionally- as well as neuroanatomically-partitioned maps might be critical in improving predictive models. Furthermore, we also know that white matter connectivity (or disconnection) plays an important role in understanding behavioural deficits (Catani and ffytche, 2005; Catani et al., 2005). A recent study has shown that damage to white matter pathways that converge into a bottleneck, for example in the posterior temporal lobe, are critical in predicting multiple behavioural deficits such as speech fluency, naming and auditory semantic decisions (Griffis et al., 2017).

The present study advances this handful of existing prediction models in two novel and important ways – namely, (a) how patients' lesions are partitioned (before they are used as predictors) and (b) in the nature and detail of what is being predicted. Our approach to both research aims was informed by a new, emerging conceptualisation of the aphasia phenotype and underlying brain systems. There is a long-standing tradition in aphasiology to categorise patients into different aphasia types according to clusters of behavioural deficits (e.g., Broca, Wernicke, conduction, etc.). These classifications provide an approximate descriptive shorthand for communicating and comparing cases across clinics/research institutions, and influencing treatment options (Horn et al., 2005). There is increasing agreement, however, that aphasia classifications have strong limitations because (a) there is considerable variability amongst patients within each category and (b) there are fuzzy boundaries between categories. Indeed, it is often difficult to place patients within a single category, leading to the diagnosis of “mixed aphasia”. An alternative approach moves away from categorisation and clustering towards considering each patient as a point in a multidimensional space, where each dimension corresponds to a primary computational-brain system (Butler et al., 2014; Chase, 2014; Halai et al., 2017). In this conceptualisation, each patient's pattern of aphasia reflects a different weighting of the impairments to these primary systems. Likewise, each language activity (e.g., naming, comprehending, repeating, etc.) is not localised to a single brain region but rather reflects the joint action of the underpinning primary systems (Patterson and Lambon Ralph, 1999; Seidenberg and McClelland, 1989; Ueno and Lambon Ralph, 2013; Ueno et al., 2011). A simple analogy is that of the arrangement of different colour hues (cf. patients) across the red, green and blue (RGB) colour space. Whilst it is possible to demarcate and label (cf. categorise) approximate areas in the space as yellow (e.g., Broca), blue (Wernicke), etc., there are in fact many different kinds of each colour and the boundaries between them are fuzzy. Likewise, when presented with individual hues it is not always obvious which colour category they fall into (e.g., teal, maroon, indigo; cf. how to categorise a patient with mixed aphasia). Thus, like aphasia classifications, colour labels provide approximate albeit limited information about the underlying graded differences. This is sufficient to communicate broad distinctions between cases (e.g., blue vs. yellow; Broca vs. Wernicke) but not finer variations (the overlapping variations of orange vs. yellow; conduction vs. Wernicke). An alternative and more precise approach is to represent each hue (patient) in terms of its position along the RGB dimensions (cf. patients' performance in terms of the underlying primary language-cognitive systems).

With sufficient breadth of assessments (to sample the full spectrum of language activities) and patient numbers, it is possible to use statistical approaches such as principal component analysis (PCA) to uncover the underlying dimensions (Lambon Ralph et al., 2002; Lambon Ralph et al., 2003). Recent applications of this approach have not only recovered the same set of orthogonal dimensions (phonology, semantics, executive skills, speech quanta) but have found that each one is associated with damage to discrete brain regions (Butler et al., 2014; Halai et al., 2017). Importantly, for the present study, very similar or identical behavioural dimensions and lesion correlates have been observed across independent studies both in patients with chronic (Lacey et al., 2017; Mirman et al., 2015a; Mirman et al., 2015b) and acute aphasia (Kümmerer et al., 2013), indicating the robustness of these core underlying factors.

The ramifications of this aphasia conceptualisation on generating prediction models are as follows. In terms of prediction targets, the ultimate aim is to predict the full behavioural profile of each patient from the neuroimaging data. Thus, rather than focussing on individual language activities, in this study we predicted each patient's scores across the full range of assessments. Given the strong tradition of using aphasia classifications, we also generated a predictive classification model but rather than focussing on pairwise discrimination between pairs of aphasia types, we required the model to discriminate *simultaneously* between all major types (thus providing a full albeit coarse-coding of the aphasic multidimensional space). Secondly, in terms of deriving the best predictors for inclusion in these models, we utilised the finding that the core underlying ‘primary’ dimensions (phonology, semantics, etc.) have been associated with discrete lesion correlates. As such, one might expect the status of each of these key regions to be a strong predictor of the patients' performance across the full range of tests. Accordingly, each patient's lesion was functionally-partitioned according to the overlap with these primary language regions and the resultant four component model was used to predict each patient's individual test scores as well as aphasia classification.

## 2 Materials and methods

### 2.1 Participants

Seventy post-stroke patients (53 males, mean age ± standard deviation [SD] = 65.21 ± 11.70 years) were recruited in the chronic stage (minimum 12 months post onset; mean = 56.6, SD = 50.17 months). A subset of cases (31/70) was the same as reported in two previous studies (Butler et al., 2014; Halai et al., 2017). The mean years in education was 12.11 (SD = 2.20). All cases were diagnosed with aphasia (using the Boston Diagnostic Aphasia Examination, BDAE), having difficulty with producing and/or understanding speech. No restrictions were placed according to aphasia type or severity (spanning from global to minimal aphasia). All subjects were right handed (premorbidly) using the Edinburgh Handedness Inventory (Oldfield, 1971), native English speakers and had only one known stroke to the left hemisphere. We excluded cases with damage to the right hemisphere or those that had multiple strokes. Data from 22 healthy age and education-matched controls (10 female, 12 male) were used to determine abnormal regions of the T1 weighted brain scan (see Neuroimaging analyses section for details). All participants gave written informed consent with ethical approval from the local ethics committee.

### 2.2 Neuropsychological assessment (dependent variables)

All participants underwent a large neuropsychological battery of tests to assess a range of language and cognitive abilities (Butler et al., 2014; Halai et al., 2017). These included subtests from the Psycholinguistic Assessments of Language Processing in Aphasia (PALPA) battery (Kay et al., 1992): auditory discrimination using non-word (PALPA 1) and word minimal pairs (PALPA 2); and immediate and delayed repetition of non-words (PALPA 8) and words (PALPA 9). Tests from the 64-item Cambridge Semantic Battery (Bozeat et al., 2000) were included: spoken and written versions of the word-to-picture matching task; Camel and Cactus Test (pictures); and the picture naming test. To increase the sensitivity to mild naming and semantic deficits we used The Boston Naming Test (BNT) (Kaplan et al., 1983) and a written 96-trial synonym judgement test (Jefferies et al., 2009). The spoken sentence comprehension task from the Comprehensive Aphasia Test (CAT) (Swinburn et al., 2005) was used to assess sentential receptive skills. Speech production deficits were assessed by coding responses to the ‘Cookie theft’ picture in the BDAE, which included tokens (TOK), mean length of utterance (MLU), type/token ratio (TTR) and words-per-minute (WPM) (see Halai et al., 2017 for more details). The additional cognitive tests included forward and backward digit span (Wechsler, 1987), the Brixton Spatial Rule Anticipation Task (Burgess and Shallice, 1997), and Raven's Coloured Progressive Matrices (Raven, 1962). Assessments were conducted with participants over several testing sessions, with the pace and number determined by the participant. The scores reflect the performance of each individual for each test and all scores were converted into percentage; if no maximum score was available for the test we used the raw score.

The neuropsychological measures were entered into a PCA with varimax rotation (SPSS 22.0). Factors with an eigenvalue exceeding 1.0 were extracted and then rotated orthogonally. Following varimax rotation, the loadings of each test allowed a clear behavioural interpretation of each factor. Individual participants' scores were obtained using the regression method for each extracted factor and used as target values in the prediction models.

### 2.3 Acquisition of neuroimaging data

High resolution structural T1-weighted Magnetic Resonance Imaging (MRI) scans were acquired on a 3.0 Tesla Philips Achieva scanner (Philips Healthcare, Best, The Netherlands) using an 8-element SENSE head coil. A T1-weighted inversion recovery sequence with 3D acquisition was employed, with the following parameters: TR (repetition time) = 9.0 ms, TE (echo time) = 3.93 ms, flip angle = 8°, 150 contiguous slices, slice thickness = 1 mm, acquired voxel size 1.0 × 1.0 × 1.0 mm^3^, matrix size 256 × 256, FOV = 256 mm, TI (inversion time) = 1150 ms, SENSE acceleration factor 2.5, total scan acquisition time = 575 s.

### 2.4 Neuroimaging analyses

**Fig. 1:**
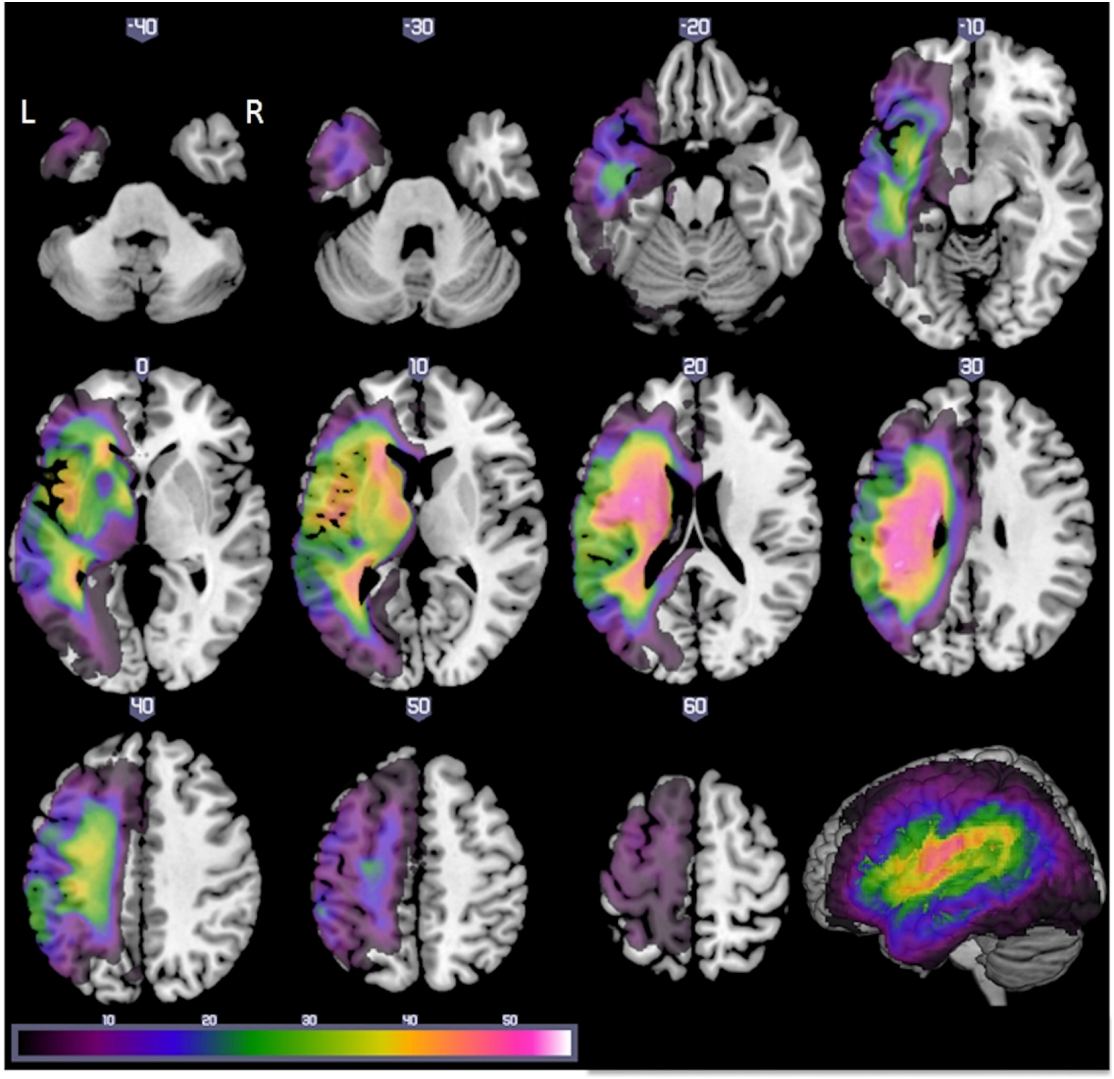
Axial slices illustrating the lesion overlap across 70 patients (threshold 1–56). Maximum overlap at [−38 −9 24] which corresponds to the central opercular cortex and left long segment and [−20 −12 26] which corresponds to the left cortico-spinal tract.

Structural MRI scans were pre-processed with Statistical Parametric Mapping software (SPM8: Wellcome Trust Centre for Neuroimaging, http://www.fil.ion.ucl.ac.uk/spm/). The images were normalised into standard Montreal Neurological Institute (MNI) space using a modified unified segmentation-normalisation procedure optimised for focal lesioned brains (Seghier et al., 2008b). Data from all participants with stroke aphasia and all healthy controls were entered into the segmentation-normalisation. This procedure combines segmentation, bias correction and spatial normalisation through the inversion of a single unified model (see Ashburner and Friston, 2005 for more details). In brief, the unified model combines tissue class (with an additional tissue class for abnormal voxels), intensity bias and non-linear warping into the same probabilistic models that are assumed to generate subject-specific images. The lesion of each patient was automatically identified using an outlier detection algorithm, compared to a group of healthy controls, based on fuzzy clustering. The default parameters were used apart from the lesion definition ‘U-threshold’, which was set to 0.5 to create a binary lesion image. We modified the U-threshold from 0.3 to 0.5 after comparing the results obtained from a sample of patients to what would be nominated as lesioned tissue by an expert neurologist. The images generated for each patient were individually checked and visually inspected with respect to the original scan, and were used to create the lesion overlap map in Fig. 1 (2 mm^3^ MNI voxel size). The binary lesion mask was then used to determine the predictor variables (see below). We selected the Seghier et al. (2008b) method as it is objective and efficient for a large sample of patients (Wilke et al., 2011), in comparison to a labour intensive hand-traced lesion mask. The method has been shown to have a DICE overlap >0.64 with manual segmentation of the lesion and >0.7 with a simulated ‘real’ lesion (where real lesions are superimposed onto healthy brains; Seghier et al., 2008b). All images were visually inspected and manually edited if required. We should note here, explicitly, that although commonly referred to as an automated ‘lesion’ segmentation method, the technique detects areas of unexpected tissue class – and, thus, identifies missing grey and white matter but also areas of augmented cerebrospinal fluid (CSF) space.

### 2.5 Predictor variables

We used the automated binary lesion mask for each subject and calculated a percentage overlap with the whole left hemisphere, giving a value of overall left hemisphere damage (referred to as the lesion volume [LV] model). We used the same binary lesion to obtain percentage lesion overlap for three critical discrete principal language-related clusters identified using a PCA voxel based correlational methodology; phonology, semantics and speech quanta (clusters taken from Halai et al., 2017), which provided three additional predictor variables (percentage of damage to each cluster based on the individuals' lesion profile) and were used in conjunction with the residual left hemisphere LV variable (referred to as the LV-PCA model). In addition to the neural predictors, we used basic demographic information as predictors including: age at testing (age), years in education (edu) and months post stroke onset (onset).

### 2.6 Prediction analysis

For the following section we used prediction-based inference to determine how well each model performed. To clarify, we used neural information from chronic T1-wieghted images to predict the patients' current chronic behavioural profile and we are not performing any predictions in the temporal sense. In order to make sure our models and predictions remained unbiased we implemented a 5-fold cross-validation procedure throughout the following analyses. Our pipeline was as follows: 1) randomly split the data into 5 folds (N = 14 per fold), 2) perform a PCA on the behavioural data on training set (4 folds), 3) determine neural correlates with PCA factor scores using voxel based correlational methodology (VBCM), 4) obtain predictor variables (as outlined above) for each cluster related to phonology, semantics and speech quanta, 5) create regression models for each neuropsychological test, and 6) use the regression to predict the holdout fold and determine the correlation between predicted data and known targets. The analyses presented in the paper are split into three parts. First, we outline the model fit (using a different combination of predictor variables) to a range of behavioural scores (measured by *adjusted* R^2^). The second analysis focused on the prediction accuracy to ‘new’ cases that were held out during the model building stage (measured by correlation). We identified the winning model by comparing the average model fit and ability to predict holdout cases across models. These evaluation measures were computed for two types of model: the first type consisted of the non-partitioned left-hemisphere lesion (LV) plus the demographic variables; and the second type of model consisted of the four-part functionally-partitioned lesion (LV-PCA predictors) plus the same demographic variables. To evaluate the statistical significance of each model (against chance) we undertook a Monte Carlo analysis. Specifically, to obtain a null distribution for each model of each behavioural test, the dependent variable (test score for each model) was randomised (N = 10,000) and the same model-fit analysis as above was performed, providing a means to test the real data against chance levels (p < 0.05). In addition, we also compared the real models directly using a Wilcoxon test (p < 0.05). In order to determine specific cases that were poorly predicted, we calculated the mean square root residual sum of squares across all test scores (sum(observed-predicted)^2^)^1/2^. Any cases that were two SD away from the mean group were considered poor predictions.

Finally, a logistic regression analysis was used to determine if the winning version of the non-partitioned LV or functionally-partitioned LV-PCA models could predict aphasia type for all subjects: 1) at a coarse level, by splitting the patients into fluent/non-fluent groups based on BDAE criteria; and 2) the specific BDAE aphasia classification. In order to obtain a null distribution for each model, the dependent variable (fluent/non-fluent or subtype code) was randomised (N = 10,000) and for each iteration a logistic regression analysis was performed. The corresponding percentage correct was recorded for each model. The threshold was set at p < 0.05 to reject the null hypothesis.

## 3 Results

### 3.1 Neuropsychological and lesion profiles

**Fig. 2:**
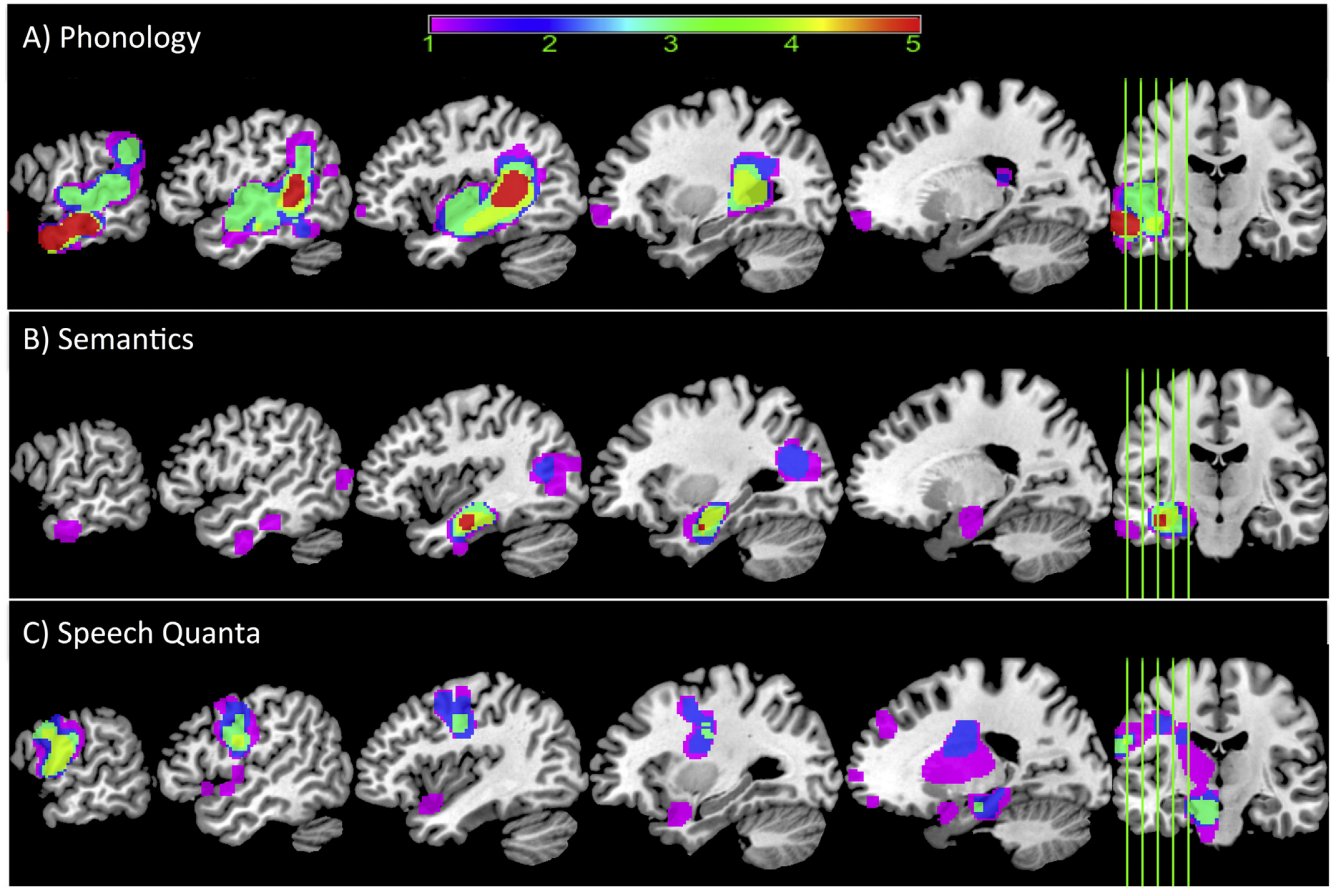
Visual overlap of neural correlates using voxel based correlational methodology (VBCM) for each fold. The figure shows how many times a voxel was present for each behavioural factor score when performing VBCM [values = 1–5 folds]. A) phonology, B) semantics, and C) fluency. Thresholded at p < 0.005 voxel height, cluster corrected using Family-Wise Error (FWE) (p < 0.05) and including lesion volume and age as a covariate.

Table 1 provides demographic details on the cases included in the study (a subset of 31 cases were reported in Butler et al., 2014; Halai et al., 2017) and overall lesion volume (note that the cases are ordered according to their Boston naming test scores). Table 2 provides a summary of the participants' scores on all neuropsychological tests (dependent variables) and is ordered according to patients' scores on the Boston naming test (note that values are displayed as integers but all decimal values used in the analyses). A lesion overlap map for all cases is provided in Fig. 1, and primarily covers the left hemisphere area supplied by the middle cerebral artery (Phan et al., 2005). The maximum number of participants who had a lesion in any one voxel was 56 (−38, −9, 24 central opercular cortex and −20 −12 26 left cortico-spinal tract). Fig. 2 shows the cluster overlap figure for each behavioural component across the 5-folds for cross-validation, which were used on each fold to determine the percentage of lesion overlap per functionally-partitioned region.

**Table 1:** Participant background information and amount of neural damage to four partitions of the brain (percentage values) (independent variables). Cases ordered according to their score on the Boston naming test.

ID	BDAE classification	Sex	Age (years)	Education (years)	Months post stroke	Lesion volume (voxels at 2 mm^3^)
1	Broca	m	85	10	46	11,393
2	Broca	m	54	13	35	18,632
3	Global	m	79	11	64	23,860
4	Global	m	72	11	42	27,054
5	Mixed nonfluent	m	67	11	44	31,317
6	Global	m	72	11	155	32,981
7	Global	m	58	13	57	33,239
8	Global	m	52	11	73	37,822
9	Mixed nonfluent	m	68	12	50	41,379
10	Conduction	m	67	17	14	6557
11	Global	m	74	11	18	19,500
12	Mixed nonfluent	m	58	13	32	14,625
13	Global	m	66	11	12	14,890
14	Broca	m	62	11	104	27,242
15	Mixed nonfluent	m	64	11	29	33,239
16	Conduction	m	67	11	13	4879
17	Mixed nonfluent	f	75	11	160	12,057
18	Mixed nonfluent	m	63	12	42	31,599
19	Conduction	m	68	11	37	4773
20	Mixed nonfluent	m	78	13	36	34,242
21	Conduction	f	77	16	34	6843
22	TSA	m	63	12	24	5822
23	Broca	m	61	11	16	3528
24	Conduction	f	46	16	21	3897
25	Broca	m	51	12	34	20,043
26	Mixed nonfluent	m	79	11	63	33,678
27	Mixed nonfluent	f	52	11	99	40,313
28	Broca	m	59	13	37	13,080
29	Mixed nonfluent	m	81	11	69	28,144
30	Broca	m	50	12	16	26,218
31	Anomia	f	53	11	47	1526
32	Broca	m	82	10	13	12,131
33	Mixed nonfluent	m	73	11	23	22,732
34	TMA	f	73	11	46	23,863
35	Anomia	m	51	13	72	22,948
36	Broca	f	48	12	16	5273
37	Anomia	f	69	19	39	9159
38	Mixed nonfluent	m	76	11	192	42,568
39	Anomia	f	51	11	66	6975
40	Broca	f	77	11	56	13,577
41	Mixed nonfluent	m	73	11	114	36,877
42	Broca	m	80	12	65	18,163
43	Anomia	m	65	10	85	6607
44	Anomia	m	44	11	40	8437
45	Anomia	m	86	9	17	8528
46	Anomia	m	59	11	34	16,433
47	Anomia	f	44	13	37	18,948
48	Anomia	m	68	11	21	3311
49	Anomia	m	75	11	11	1481
50	Anomia	m	87	12	35	8238
51	Anomia	m	66	11	126	15,492
52	Mixed nonfluent	m	67	11	120	26,097
53	Conduction	m	84	9	35	7854
54	Anomia	m	85	10	69	21,489
55	Mixed nonfluent	f	67	14	176	26,283
56	Anomia	m	65	17	25	4806
57	Anomia	m	68	11	14	8788
58	Anomia	m	52	17	33	11,915
59	TMA	m	76	11	116	11,239
60	Anomia	m	45	11	25	10,409
61	Anomia	m	50	19	16	4538
62	Anomia	f	58	11	278	12,699
63	Anomia	m	67	11	60	10,073
64	Broca	m	58	11	135	18,392
65	Anomia	m	56	16	17	6974
66	Anomia	f	73	11	89	8921
67	Anomia	f	68	16	22	8118
68	Anomia	f	52	12	76	9767
69	Anomia	f	43	16	15	175
70	Anomia	m	63	12	10	18,639

**Table 2:** Participant scores on behavioural assessment battery and speech production measures (dependent variables). All scores have been converted into percentage and rounded to an integer, except for TTR, WPM, TOK and MLU. Abbreviations: Minimal pairs non-word (PALPA 1), Minimal pairs word (PALPA 2), non-word immediate repetition (PALPA 8 I), non-word delayed repetition (PALPA 8 D), word immediate repetition (PALPA 9 I), word delayed repetition (PALPA 8 D), Cambridge naming test (CNT), Boston naming test (BNT), forward digit span (Digit F), backward digit span (Digit B), spoken sentence comprehension from comprehensive aphasia test (CAT spoken), spoken word-picture matching (sWPM), written word-picture matching (wWPM), type/token ratio (TTR), camel and cactus picture form (CCTp), 96-synonym judgement task (Synon), words-per-minute (WPM), speech tokens (TOK) and mean length of utterances (MLU).

ID	PALPA 8 I	PALPA 8 D	PALPA 9 I	PALPA 9 D	CNT	BNT	Digit F	Digit B	CAT-spoken	sWPM	wWPM	TRR	CCTp	Synon	PALPA 1	PALPA 2	Ravens	Brixton	WPM	TOK	MLU
1	0	0	4	0	2	0	50	43	84	95	92	0.75	89	82	75	65	81	51	3	4	2
2	0	0	0	0	5	0	38	43	75	100	98	0.48	83	78	92	97	89	76	106	146	19
3	0	0	0	0	0	0	25	0	25	33	28	0.00	34	48	76	71	47	33	0	0	0
4	0	0	0	0	5	0	0	0	25	78	91	0.82	73	73	49	54	67	40	21	33	5
5	0	0	5	0	2	0	25	0	50	78	56	1.00	59	49	92	96	64	35	1	1	0
6	0	0	0	0	0	0	0	0	34	88	61	0.00	44	52	54	50	39	24	0	0	0
7	0	0	1	0	0	0	25	0	47	78	94	0.00	67	75	82	78	92	92	0	0	0
8	0	0	0	0	0	0	0	0	34	59	66	0.00	63	52	85	86	89	53	0	0	0
9	0	0	38	0	0	0	25	0	13	58	31	0.47	53	49	22	53	31	38	87	32	8
10	3	3	21	5	3	2	25	29	63	97	97	0.51	89	90	96	96	83	62	94	203	19
11	0	0	36	0	6	2	25	0	50	77	59	0.33	61	43	56	53	47	47	18	6	2
12	0	0	6	0	5	2	0	0	28	92	98	0.39	78	69	75	78	89	62	16	18	3
13	13	3	28	0	2	2	0	0	28	67	53	0.40	69	57	43	54	61	33	20	10	3
14	3	0	5	1	3	2	0	0	31	92	100	0.55	84	78	65	75	92	38	73	29	7
15	53	20	89	39	14	3	38	0	47	63	92	0.29	66	47	97	86	83	65	33	58	5
16	0	3	14	8	6	5	25	29	59	100	98	0.65	89	70	93	99	81	75	110	55	9
17	0	0	0	0	5	5	25	0	56	100	95	0.39	78	74	74	85	50	35	15	46	5
18	70	30	85	84	8	8	38	29	31	64	77	0.33	83	59	88	58	86	40	24	120	9
19	0	3	35	23	16	8	38	43	88	100	100	0.61	84	72	89	82	69	55	44	66	10
20	23	7	44	35	39	10	75	43	34	100	97	0.91	73	66	96	93	78	60	5	11	1
21	13	3	45	41	20	12	25	29	50	97	100	0.53	84	79	78	86	81	42	68	203	13
22	73	80	94	95	31	13	100	57	84	72	67	0.75	69	84	94	96	86	53	97	81	12
23	23	17	49	33	42	15	63	43	88	100	100	0.80	91	89	96	94	83	65	17	25	3
24	0	0	39	24	34	15	38	0	72	100	100	0.55	92	90	79	81	97	80	27	38	3
25	33	3	56	26	42	15	0	0	47	94	95	0.54	84	81	72	96	100	76	20	61	7
26	17	3	63	33	25	17	25	0	44	86	61	0.67	53	46	47	43	61	35	8	12	5
27	33	0	70	19	44	20	25	0	69	89	97	1.00	84	77	74	64	92	51	8	11	2
28	10	3	51	43	55	23	25	29	56	98	100	0.68	92	82	89	86	89	44	30	31	7
29	37	30	55	41	39	25	38	29	50	92	75	0.57	52	61	47	64	61	44	8	14	3
30	93	63	100	81	69	32	38	29	66	97	95	0.49	73	75	99	97	92	58	13	70	4
31	67	37	81	79	83	38	50	29	75	100	98	0.68	91	83	93	96	67	47	57	56	15
32	33	17	65	66	66	38	88	29	91	97	98	0.83	81	85	86	82	89	62	17	18	8
33	27	17	50	61	47	38	38	29	13	97	94	0.18	59	57	99	99	39	58	7	33	3
34	57	43	85	91	61	38	75	0	88	100	100	0.64	84	83	81	94	81	51	20	25	5
35	27	13	86	64	77	42	38	29	75	95	98	0.52	80	75	81	88	89	89	56	122	17
36	40	57	73	69	67	43	63	57	94	100	98	0.81	95	92	97	97	100	82	20	31	10
37	50	47	90	89	89	43	50	57	81	100	100	0.47	95	97	75	93	100	65	106	315	20
38	30	3	75	65	63	47	50	29	59	92	98	0.65	77	81	75	78	83	67	43	34	7
39	53	10	94	41	88	50	38	29	63	100	97	0.72	92	78	90	96	89	44	49	47	10
40	23	13	58	55	53	50	75	43	69	97	98	0.60	86	82	96	93	64	31	25	25	4
41	10	13	65	55	59	52	25	29	63	92	98	0.63	66	70	92	93	47	31	20	19	3
42	37	63	90	91	72	53	63	43	75	98	98	0.79	83	76	75	86	78	44	33	28	8
43	47	30	71	68	78	55	63	43	84	100	100	0.59	88	88	88	92	92	71	55	69	12
44	100	90	100	100	89	55	50	57	88	100	100	0.66	91	91	99	97	97	69	56	56	15
45	30	10	76	71	55	55	63	43	75	98	98	0.57	75	95	79	76	53	71	98	122	17
46	43	47	95	95	72	57	38	29	78	98	100	0.52	94	90	99	96	97	78	56	94	12
47	60	40	93	89	84	60	50	29	84	100	100	0.61	91	88	96	97	92	76	24	38	7
48	83	70	96	96	84	62	88	43	78	100	100	0.73	84	79	96	96	78	65	93	74	13
49	63	37	86	81	75	63	63	29	84	94	91	0.80	78	75	76	89	86	60	57	35	6
50	37	23	83	74	86	63	63	43	56	98	98	0.74	53	94	51	81	42	44	58	54	11
51	83	83	100	99	81	63	50	57	100	100	100	0.68	81	82	93	96	94	76	109	80	11
52	37	40	85	79	80	63	38	0	78	100	98	0.65	88	64	90	96	81	62	64	34	8
53	13	17	61	54	84	63	63	57	63	97	98	0.78	95	94	57	60	81	73	60	40	8
54	37	23	84	80	66	65	50	43	38	94	94	0.79	86	85	78	76	75	25	35	63	12
55	53	37	70	81	72	65	25	0	56	95	94	0.71	91	84	82	89	39	42	64	63	13
56	83	53	100	100	81	67	63	57	72	98	100	0.58	91	95	94	99	100	80	109	125	13
57	87	80	100	96	95	67	63	57	78	98	94	0.68	73	83	93	94	67	67	51	38	8
58	60	10	74	69	75	72	38	0	56	98	98	0.74	98	96	81	93	92	51	33	38	7
59	57	33	89	91	84	73	50	29	88	100	100	0.87	89	94	79	78	72	73	48	23	14
60	70	67	88	73	88	73	50	29	94	100	100	0.90	91	88	99	99	97	76	44	48	15
61	50	37	85	90	78	77	63	43	100	100	100	0.51	92	96	88	97	94	75	49	94	14
62	60	57	95	94	84	77	63	0	81	98	98	0.87	83	89	82	92	83	64	37	23	5
63	57	50	83	89	81	78	88	57	91	97	100	0.65	88	93	96	97	89	76	91	82	7
64	73	83	78	88	88	78	100	100	88	100	100	0.70	75	90	100	99	75	56	18	30	7
65	80	47	91	99	88	78	50	43	94	95	97	0.55	92	96	86	93	83	69	48	116	17
66	93	67	96	99	97	80	63	57	72	100	100	0.60	94	92	90	96	83	51	70	65	10
67	90	80	98	95	94	85	75	71	97	100	100	0.70	94	94	90	97	97	67	98	87	17
68	90	90	100	99	94	88	88	86	84	100	100	0.75	80	94	92	100	92	60	212	60	12
69	87	80	100	100	94	88	63	57	91	98	98	0.91	84	86	99	99	94	71	69	22	10
70	93	83	99	93	95	95	63	29	69	98	100	0.67	70	89	88	99	50	53	53	51	11

In order to evaluate the utility of the models the results are split into three sections. First, we summarise the *adjusted* R^2^ values for each model across behavioural tests. Secondly, we determine which model had the best predictive power by comparing the correlation for holdout folds for each model across behavioural tests. Finally, we show how well the winning models can classify patients based on fluent/non-fluent membership and specific BDAE classification using logistic regression.

### 3.2 Best fit to data (adjusted R^2^)

**Fig. 3:**
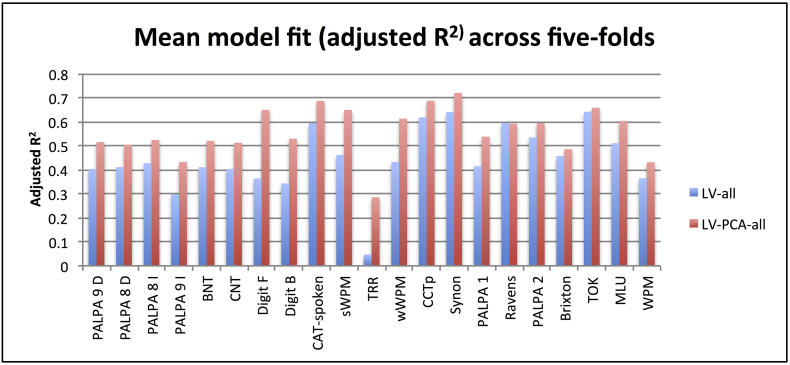
Model fit (adjusted R^2^ values) across tests for the best model from the non-partitioned (LV-all) and functionally-partitioned lesion (LV-PCA-all) model groups. Abbreviations: Minimal pairs non-word (PALPA 1), Minimal pairs word (PALPA 2), non-word immediate repetition (PALPA 8 I), non-word delayed repetition (PALPA 8 D), word immediate repetition (PALPA 9 I), word delayed repetition (PALPA 8 D), Cambridge naming test (CNT), Boston naming test (BNT), forward digit span (Digit F), backward digit span (Digit B), spoken sentence comprehension from comprehensive aphasia test (CAT spoken), spoken word-picture matching (sWPM), written word-picture matching (wWPM), type/token ratio (TTR), camel and cactus picture form (CCTp), 96-synonym judgement task (Synon), words-per-minute (WPM), speech tokens (TOK) and mean length of utterances (MLU).

For a graphical depiction of the adjusted R^2^ values for all tests, see Fig. 3. Table 3 shows the mean adjusted R^2^ values and highlights significant differences between all model pairings. For simplicity, the Figure only shows the adjusted R^2^ values for LV-all and LV-PCA-all as these models were approximately the two best models. Overall, the fit to data for both LV and LV-PCA models (and the variant models with demographic information) was significantly better than chance (p < 0.05) for almost every behavioural test (see Supplementary Table A1 for scores on each behavioural test, values marked in bold indicate non-significant models). Importantly, the average adjusted R^2^ across all tests was significantly higher for the functionally-partitioned LV-PCA model compared to the LV model (0.27 and 0.15, respectively) (Wilcoxon Test: Z = 4.015, p < 0.001). There were four language assessments that there were problematic for some models: A) The LV-age and LV-edu models did not significantly fit to immediate word repetition and Boston naming test scores; B) The LV and LV-ons models did not significantly fit to the Brixton score; and C) The LV, LV-age, LV-edu, LV-ons, LV-all models did not significantly fit to type/token ratio (although it should be noted that this assessment had the lowest adjusted R^2^ values overall for all models). Considering the non-partitioned LV models, the best-fitting (adjusted R^2^) models when adding demographic variables were LV-ons (0.17) and LV-all (0.22). The LV-all model was significantly better than all other LV models (p's < 0.016) except LV-ons (Wilcoxon Test: Z = 1.616, p = 0.106). The LV-ons was only significantly better than LV (Wilcoxon Test: Z = 2.311, p = 0.021). Considering the functionally-partitioned lesion (LV-PCA) models, the best-fitting (adjusted R^2^) models when adding demographic variables were LV-PCA-all (0.323) and LV-age (0.311). The LV-PCA-all model was significantly better than LV-PCA, LV-PCA-edu and LV-PCA-ons (p's < 0.03) but not different to LV-PCA-age (Wilcoxon Test: Z = 0.539, p = 0.590). The LV-PCA-age model was not different to LV-PCA-edu but was trending towards significance against LV-PCA and LV-PCA-ons (Wilcoxon Test: Z = 1.894, p = 0.058).

**Table 3:** Comparing Best model fit (adjusted R^2^) and predictive power (predictive R^2^) across all models using a Wilcoxon repeated measures test. Mean values for adjusted and predicted R^2^ are given in the second column, whilst significant differences between each pair-wise comparison (p < 0.05) is shown in **bold**.

Adjusted R^2^	Mean	LV	LV-age	LV-edu	LV-ons	LV-ons	LV-PCA	LV-PCA-age	LV-PCA-edu	LV-PCA-ons	LV-PCA-all
LV	0.150	1	0.159	0.566	**0.021**	**<0.001**	**<0.001**	**<0.001**	**<0.001**	**<0.001**	**<0.001**
LV-age	0.185		1	0.099	0.958	**0.016**	**0.013**	**<0.001**	**0.004**	**0.012**	**<0.001**
LV-edu	0.166			1	0.339	**0.002**	**0.001**	**0.001**	**<0.001**	**0.001**	**<0.001**
LV-ons	0.171				1	0.106	**<0.001**	**<0.001**	**<0.001**	**<0.001**	**<0.001**
LV-ons	0.217					1	0.085	**0.001**	**0.010**	0.092	**<0.001**
LV-PCA	0.269						1	0.058	0.455	0.958	**0.007**
LV-PCA-age	0.311							1	0.122	0.058	0.590
LV-PCA-edu	0.286								1	0.455	**0.021**
LV-PCA-ons	0.270									1	**0.030**
LV-PCA-all	0.323										1


Holdout correlation	Mean	LV	LV-age	LV-edu	LV-ons	LV-ons	LV-PCA	LV-PCA-age	LV-PCA-edu	LV-PCA-ons	LV-PCA-all
LV	0.320	1	**<0.001**	0.274	0.375	0.063	**0.002**	**<0.001**	**<0.001**	**0.012**	**<0.001**
LV-age	0.385		1	**0.013**	**0.011**	0.434	0.543	**<0.001**	0.149	0.903	**0.001**
LV-edu	0.339			1	0.159	0.114	**0.021**	**<0.001**	**0.001**	0.122	**<0.001**
LV-ons	0.302				1	**0.008**	**0.001**	**<0.001**	**<0.001**	**0.001**	**<0.001**
LV-ons	0.369					1	0.411	**0.001**	0.099	0.715	**<0.001**
LV-PCA	0.406						1	**0.002**	**0.004**	0.058	**0.009**
LV-PCA-age	0.484							1	**0.023**	**0.001**	**0.046**
LV-PCA-edu	0.440								1	**0.003**	0.131
LV-PCA-ons	0.391									1	**0.001**
LV-PCA-all	0.478										1

In order to compare the relative power of these models further, we summed the number of assessment tests for which each model had the best fit for the four models identified above. The functionally-partitioned models were the best for the vast majority of individual tests: LV-ons (0/21), LV-all (0/21), LV-PCA-age (13/21) and LV-PCA-all (8/21).

### 3.3 Predictive power (predicted R^2^)

The non-partitioned lesion-only (LV) model had significantly greater correlation values between predicted and observed than chance levels for all bar five measures (PALPA 8 immediate, PALPA 9 immediate, PALPA 9 delayed, TTR, and the Brixton spatial anticipation test). The functionally-partitioned LV-PCA only model had significantly greater correlation values between predicted and observed scores than chance levels for all bar two measures (PALPA 8 immediate and PALPA 9 immediate). It should be noted that most models failed to accuracy predict PALPA 8 immediate, PALPA 9 immediate and TTR (see Supplementary Table A2 for details on all models, values marked in bold reflect non-significant models). Table 3 shows the mean correlation between the predicted and observed values across holdout folds for all models. Overall, the correlations were significantly higher for the LV-PCA model than the LV model (0.41 vs. 0.32, respectively; Wilcoxon Test: Z = 3.146, p = 0.002). We report the correlation values for: LV-age, LV-all, LV-PCA-age and LV-PCA-all as these produced the best models. There were a small number of behavioural tests that were not predicted above chance-level based on these four models: the LV-age model failed to generate better than chance predictions for PALPA 9 (delayed) and TTR measures; the LV-all model failed for PALPA 9 (immediate and delayed), PALPA 8 (immediate), forward digit span, and TTR; the LV-PCA-all model failed on PALPA 9 (immediate) and TTR; whilst the LV-PCA-age model did not fail on any measure.

**Fig. 4:**
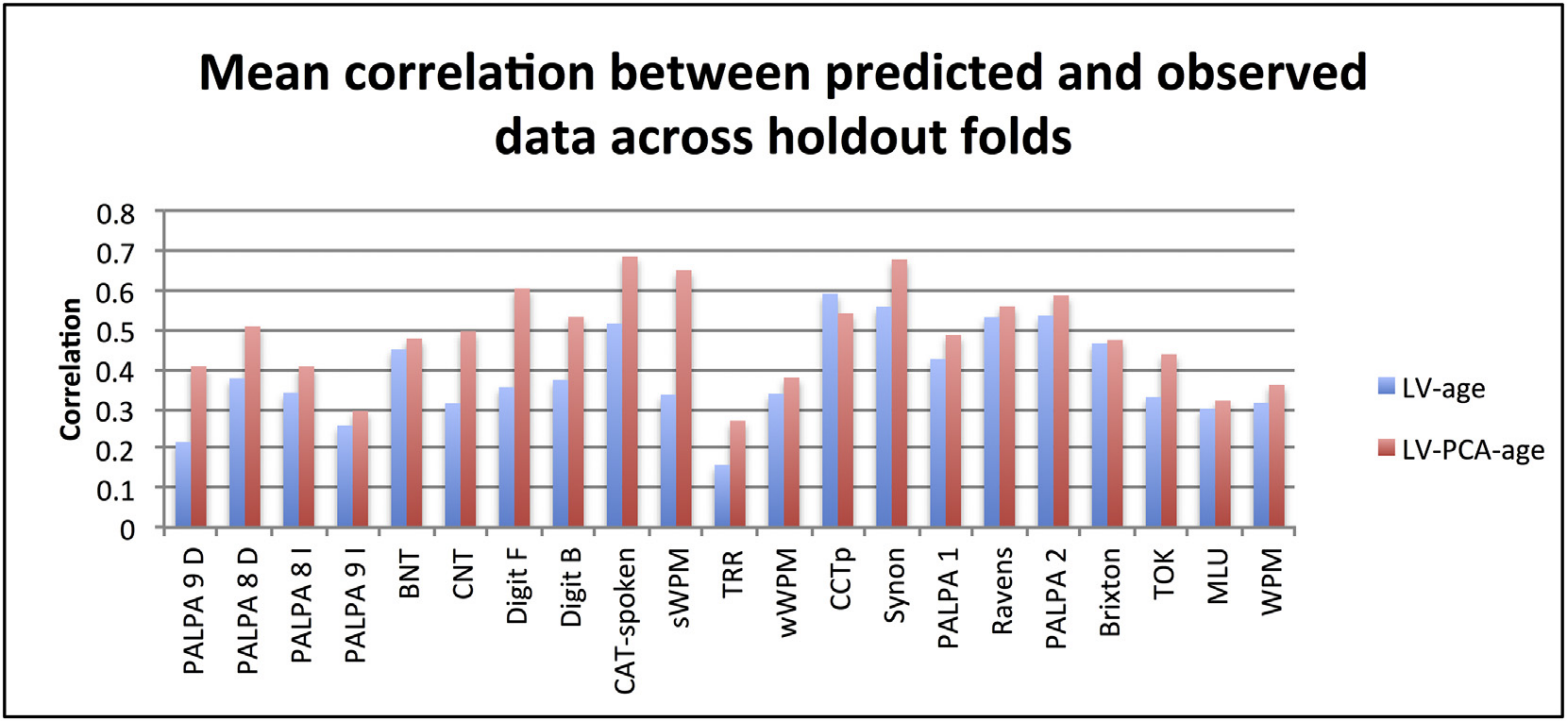
Mean correlation for predicted and observed values across tests for the best model from the non-partitioned (LV-age) and functionally-partitioned lesion (LV-PCA-age) model groups. Abbreviations: Minimal pairs non-word (PALPA 1), Minimal pairs word (PALPA 2), non-word immediate repetition (PALPA 8 I), non-word delayed repetition (PALPA 8 D), word immediate repetition (PALPA 9 I), word delayed repetition (PALPA 8 D), Cambridge naming test (CNT), Boston naming test (BNT), forward digit span (Digit F), backward digit span (Digit B), spoken sentence comprehension from comprehensive aphasia test (CAT spoken), spoken word-picture matching (sWPM), written word-picture matching (wWPM), type/token ratio (TTR), camel and cactus picture form (CCTp), 96-synonym judgement task (Synon), words-per-minute (WPM), speech tokens (TOK) and mean length of utterances (MLU).

For the non-partitioned lesion (LV) models, the highest mean correlation belonged to LV-age but this was not significantly greater than LV-all (0.385 vs. 0.369, respectively; Wilcoxon Test: Z = 0.782, p = 0.434). For the functionally-partitioned lesion (LV-PCA) models, LV-PCA-age did produce significantly higher values than LV-PCA-all (0.484 vs. 0.478; Wilcoxon Test: Z = 1.999, p < 0.046). Directly comparing the LV-age and LV-PCA-age models, showed the latter model had significantly higher correlations (Wilcoxon Test: Z = 3.736, p < 0.001). Therefore in both model groups, adding age to the neural information produced significantly higher correlation values than neural information alone, as well as all demographic variables combined. Fig. 4 shows the mean correlation values for all tests based on the LV-age and LV-PCA-age models.

We identified two cases for which the predictive model performed poorly (2/70 or 2.86%) and had residual scores more than two SD from the mean group (Case 37 and 40). On closer inspection the model was poor at repetition and picture naming performance for case 37 and speech tokens for case 40. The repetition and picture naming performance for case 37 was floor for all tests, however the lesion profile of the patient suggests that frontal regions related to speech output were disproportionally affected compared to regions related to phonological processes (proportion of neural damage: LV 9.1%, phonology cluster 7.7%, semantic cluster 7% and speech quanta cluster 50.6%). The discrepancy for case 40 was solely based on underestimating the number of tokens produced (predicted 72 vs. 315 observed).

As with model fit (adjusted R^2^), we investigated the number of tests for which each model was the best performer. The results were as followed: LV-age (1/21), LV-all (1/21), LV-PCA-age (13/21) and LV-PCA-all (6/21).

**Fig. A1:**
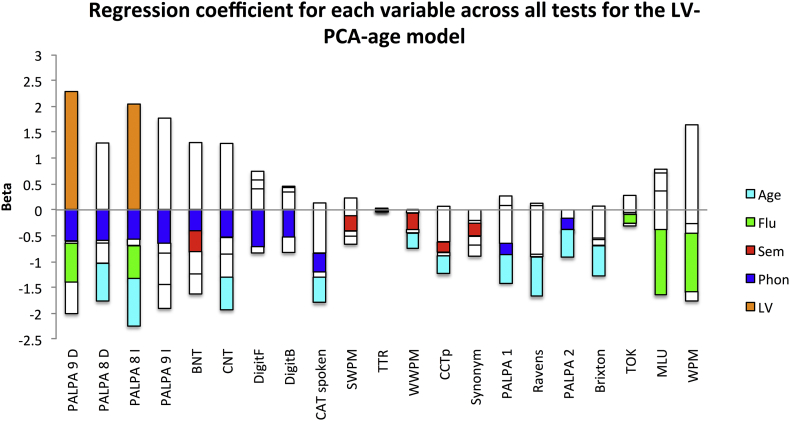
A graphical depiction of the regression coefficients for the LV-PCA-age model across all neuropsychological tests. The beta weights are plotted on the x-axis and the bars are coloured coded according to the independent variable: Lesion volume (LV) - orange; phonology – blue; semantics – red; fluency – green; and age – cyan. Abbreviations: Minimal pairs non-word (PALPA 1), Minimal pairs word (PALPA 2), non-word immediate repetition (PALPA 8 I), non-word delayed repetition (PALPA 8 D), word immediate repetition (PALPA 9 I), word delayed repetition (PALPA 8 D), Cambridge naming test (CNT), Boston naming test (BNT), forward digit span (DigitF), backward digit span (DigitB), spoken sentence comprehension from comprehensive aphasia test (CAT spoken), spoken word-picture matching (sWPM), written word-picture matching (wWPM), type/token ratio (TTR), camel and cactus picture form (CCTp), 96-synonym judgement task (Synon), words-per-minute (WPM), speech tokens (TOK) and mean length of utterances (MLU).

Finally, in order to determine the distribution and significance of the betas in the winning model we determined the model fit including all participants across all test for the LV-PCA-age model (see Supplementary Fig. A1).

### 3.4 Aphasia classification

Overall, we deemed the LV-age and LV-PCA-age models to be the winning models in each group of models based on the model fit and predictive capabilities. The following classification analyses were split into two stages: fluent/non-fluent and specific subtypes. The results for the binary classification of fluent vs. non-fluent aphasia were significant for LV-age (80% accuracy, p = 0.043) and LV-PCA-age (88.6% accuracy, p = 0.003) when compared to distribution obtain using permutation tests (the mean chance level was 49.75% and 49.08%, respectively). The difference between the two models was at a trend, in favour of the LV-PCA-age model (Wilcoxon Test: Z = 1.90, p = 0.06). The LV-PCA-age model classified 87.9% (29/33) fluent cases and 89.2% (33/37) non-fluent cases correctly, compared to the LV-age model which achieved 84.8% (28/33) and 75.7% (28/37), respectively. The coefficients in the logistic regression for the LV-PCA-age model were as follows: LV (0.048), phonology (−0.044), semantic (−0.013), fluency (−0.096) and age (−0.036), where only fluency was significant (p = 0.003 and remaining betas had p > 0.092).

Secondly, we used a multinomial logistic regression to determine how well each model could classify patients simultaneously into the seven BDAE subtypes. The models were both significantly better than chance determined by permutation testing: LV-age (54.3% accuracy) and LV-PCA-age (68.6%) where the mean chance levels were 36.2% and 32.9%, respectively. The difference between the two models was significant, in favour of the LV-PCA-age model (Wilcoxon Test: Z = 2.36, p = 0.018). The LV-PCA-age model correctly classified 77.8% anomia, 58.3% Broca, 33.33% conduction, 42.9% global, 86.7% mixed non-fluent, 50% TMA and 100% TSA. In comparison the LV-age model correctly classified 85.2% anomia, 8.3% Broca, 16.7% conduction and 86.7% mixed non-fluent cases (remaining aphasia types, all 0%). Overall, the LV-PCA-age model outperformed the LV-age model in predicting fluent/non-fluent status and BDAE classifications.

## 4 Discussion

Our understanding of how our speech and language capabilities are organised in the brain has vastly improved over the past decade (reflected not only in a large number of published papers but also in vibrant dedicated, international learned societies such as the Society for the Neurobiology of Language: http://www.neurolang.org/). It is, therefore, both timely and critical to use this accumulated knowledge and expertise to address critical research challenges, including the ability to predict the behavioural deficits experienced after brain damage not only to validate the theoretical models but also to provide improved care and clinical management. The approach taken in this study to tackle these aims was based on a new, emerging conceptualisation of the aphasia phenotype and underlying brain systems (Butler et al., 2014; Chase, 2014; Halai et al., 2017). Specifically, rather than relating each language activity (e.g., repetition, naming, comprehension, etc.) singly to the underlying neural systems, the varying aphasia phenotype (both severity and type) is hypothesised to reflect graded differences in the level of damage to a set of primary neurocognitive systems (Patterson and Lambon Ralph, 1999; Seidenberg and McClelland, 1989; Ueno and Lambon Ralph, 2013; Ueno et al., 2011) and their interaction. By utilising a combination of principal component analysis on a large and detailed behavioural dataset and voxel-symptom lesion mapping, previous studies have shown that (a) the variable aphasia phenotype can be considered in terms of graded differences along a set of statistically-independent (orthogonal) dimensions (e.g., semantics, phonology, speech quanta, executive skill (Butler et al., 2014; Halai et al., 2017; Kümmerer et al., 2013; Lacey et al., 2017; Mirman et al., 2015a; Mirman et al., 2015b)) and (b) that each of these factors is associated with increased lesion likelihood in discrete brain regions (e.g., semantics – anterior temporal lobe; phonology – posterior superior temporal gyrus/inferior supramarginal gyrus, etc.).

Based on this new, evolving conceptualisation of aphasia, the current study tested a prediction model that had two important novel features: (a) as well as predicting aphasia type or individual features of aphasic performance (as done in the small handful of previous studies (Hope et al., 2015; Hope et al., 2013; Saur et al., 2010; Yourganov et al., 2015), we targeted the full spectrum of aphasia by predicting aphasia type as well as the full range of behavioural test scores; (b) the patients' lesions were functionally-partitioned before using them as predictors. Specifically, we demonstrated that the functionally-partitioned (LV-PCA) model outperformed a more general model that only incorporated the overall lesion volume (LV model), both in terms of percentage variance explained of the training data and correlation of predictions on left out cases. By adding age to the functionally-partitioned model, we further improved the explanatory and predictive power of the model for the majority of behavioural tests. Furthermore, this combined (LV-PCA plus age) model was better at classifying participants into aphasia subtypes compared to the unpartitioned lesion plus age (LV-age) model. Indeed, the LV-PCA-age model was able to classify a broad range of patient subtypes. The success of the LV-PCA-age model suggests that: 1) the a priori functional partitions (identified in previous studies using a combination of PCA-decomposition of detailed behavioural data and voxel-lesion symptom mapping) are suitable to capture variance across a wide range of aphasia patients; and 2) age proved to be the best demographic variable across the range of tests.

Previous studies that have investigated the utility neural information for predict behavioural outcomes/deficits have had mixed results. Earlier reports suggested that neural information does not add significantly to predictions (Hand et al., 2006; Johnston et al., 2002; Johnston et al., 2007; Lazar et al., 2008; Willmes and Poeck, 1993). Our results align with the more contemporary studies that have found significant improvements in predictions when using neural lesion information (Hope et al., 2013; Saur et al., 2010; Schiemanck et al., 2006; Thijs et al., 2000; Yourganov et al., 2015). Currently, the existing literature has either focused on differentiating between pairs of aphasia subtypes (i.e. Yourganov et al., 2015) or has targeted individual, important tests scores (Hope et al., 2013). There has been only one study that has predicted the subtests within the CAT using anatomical regions defined in pre-existing atlases (Hope et al., 2015). Our investigation suggests that the full range of aphasia subtypes can be predicted (to provide a ‘coarse’ picture of the nature of each patient's type and severity of aphasia; see Introduction section for the limitations of these measures) and can also predict a broad spectrum of individual assessment scores (to provide a detailed picture of each patient's phenotype).

A critical characteristic of the prediction model was its use of functionally-derived partitioning of the patients' lesions rather than the use of anatomical parcellations (e.g. Hope et al., 2013; Yourganov et al., 2015). It is important to note that the lesion correlations across 5-folds produced strikingly stable results, suggesting that the core areas identified are highly reproducible. This alternative approach resonates with a previous study by Saur et al. (2010) which found that including the level of signal from functionally-focused regions-of-interest in patients' acute fMRI scans, enhanced binary predictions of language outcome and improvement (rather than aphasia types or assessment profiles). Although we did not use fMRI data in the current study (instead deriving the functional partitioning from a combined PCA-lesion mapping approach (Halai et al., 2017)), the fact that both studies found considerable improvements in prediction power suggests that functionally-related information may be a critical ingredient for successful prediction models.

In clinical terms, the prediction accuracy of aphasia classification achieved here was very good and thus high enough to begin to contemplate how this model might be used in clinical management, including predicting language-cognitive abilities in the chronic stage from scans collected in the acute or sub-acute phase, to guide intervention plans and to stratify patients – in short, a form of ‘neurocognitive’ precision medicine. We stress that the current models were built and tested on chronic neural and behavioural data and were not tested in the temporal sense (acute to chronic) – which can be explored in future studies (although this type of prediction has additional barriers; see Karnath and Rennig, 2017). In contrast, whilst predictions of the specific scores across the full test battery are statistically reliable and better than lesion-only models, further improvements of the models are required before they could be used clinically.

Future studies can explore how to improve the predictive power of these fine-grained prediction models. It seems likely that these explorations will fall into three classes: (i) *more data*: like our own investigation, most studies to date have used structural (T1/T2) neuroimaging to predict performance – which is important given that routine clinical scanning often only includes this type of scan. The inclusion of other imaging modalities, such as fMRI (Saur et al., 2010), white-matter connectivity (e.g. Corbetta et al., 2015; Griffis et al., 2017; Saur et al., 2008) and functional-connectivity, might improve prediction models, especially when used together (which will require sophisticated methods for combined analyses of multimodal imaging data (Calhoun and Sui, 2016)). Furthermore, and perhaps critically, these additional imaging measures might provide critical insights about post-stroke functional reorganisation which is unlikely to be reflected in the core lesion itself but rather from changes in functional activation and connectivity. (ii) *More predictors*: as well as developing the precision of each existing predictor, it is likely that the models will be improved by increasing the range (in terms of additional areas) and type (in terms of modality) of neuroimaging predictors. These will include other aspects of language processing (e.g., perhaps the differentiation of receptive and expressive phonological abilities (Schwartz et al., 2012; Schwartz et al., 2009)), as well as non-language functions (e.g., executive abilities). The inclusion of non-language primary systems such as executive skill may be important given that it has been shown to vary across the aphasia population, is engaged by patients with better recovery, predicts response to therapy and forms a critical part of the aphasia phenotype (Brownsett et al., 2014; Butler et al., 2014; Fillingham et al., 2005; Halai et al., 2017). (iii) *Individual differences*: whilst some previous studies have predicted dichotomies (e.g., contrastive aphasias or good/bad outcomes), some like the present study have attempted to predict the individual differences in performance (e.g. Hope et al., 2015; Hope et al., 2013). There are, however, other sources of individual differences which may be important. First, there are premorbid differences in how individuals achieve each language activity, in terms of the reliance on different parts and connections within the neurocognitive network that underpins the behavioural activity. Indeed, the multiple parts and connections in these networks may promote (individually-varying) robustness to task complexity and damage (a notion capture by the mathematical term, *degeneracy* (Price and Friston, 2002)). Recent explorations using fMRI in healthy participants suggest that it may be possible to map and understand the limits of these premorbid differences and, with transcranial magnetic stimulation, to understand the likely impact following brain damage (Hoffman et al., 2015; Kherif et al., 2008; Seghier et al., 2008a; Woollams et al., 2016). Secondly, there are clearly individual differences in the level and type of recovery after brain damage. Prediction models will be improved, therefore, not only by identifying global factors that modulate the overall level of recovery (which might, perhaps, include age and domain-general mechanisms such as multi-demand executive skills, see above) but also the limits on how far key neurocognitive networks can re-distribute function after partial damage (Keidel et al., 2010; Ueno and Lambon Ralph, 2013; Ueno et al., 2011; Welbourne and Lambon Ralph, 2007; Welbourne et al., 2011).

The following are the supplementary data related to this article.

Supplementary tables Image 1
