# Supplementary material for: Predicting the pattern and severity of chronic post-stroke language deficits from functionally-partitioned structural lesions
Source: Neuroimage Clin. 2018 Mar 16;19:1–13. doi: 10.1016/j.nicl.2018.03.011 (PMC6051318; doi:10.1016/j.nicl.2018.03.011)
Supplement: Supplementary file 1 — Supplementary tables [file mmc1.docx]

**Supplementary materials.**

**Tables**

Table A1. Best model fit (adjusted R^2^) for each behavioural test using various models. The lesion volume only (LV) and lesion volume plus principal component defined regions (LV-PCA) are shown alone and along with various demographic information. Bold text highlight models that were not significant above chance (p>0.05). Abbreviations: years in education (edu), months post onset (onset), Minimal pairs non-word (PALPA 1), Minimal pairs word (PALPA 2), non-word immediate repetition (PALPA 8 I), non-word delayed repetition (PALPA 8 D), word immediate repetition (PALPA 9 I), word delayed repetition (PALPA 8 D), Cambridge naming test (CNT), Boston naming test (BNT), forward digit span (Digit F), backward digit span (Digit B), spoken sentence comprehension from comprehensive aphasia test (CAT spoken), spoken word-picture matching (sWPM), written word-picture matching (wWPM), type/token ratio (TTR), camel and cactus picture form (CCTp), 96-synonym judgement task (Synon), words-per-minute (WPM), speech tokens (TOK) and mean length of utterances (MLU).

| Adjusted R2 | PALPA 8 I | PALPA 8 D | PALPA 9 I | PALPA 9 D | CNT | BNT | Digit F | Digit B | CAT-spoken | sWPM | wWPM | TRR | CCTp | Synon | PALPA 1 | PALPA 2 | Rav-ens | Brix-ton | WPM | TOK | MLU |
| --- | --- | --- | --- | --- | --- | --- | --- | --- | --- | --- | --- | --- | --- | --- | --- | --- | --- | --- | --- | --- | --- |
| LV | 0.13 | 0.13 | 0.08 | 0.13 | 0.11 | 0.09 | 0.12 | 0.16 | 0.30 | 0.21 | 0.16 | **0.00** | 0.28 | 0.32 | 0.09 | 0.15 | 0.10 | **0.07** | 0.15 | 0.15 | 0.23 |
| LV-age | 0.17 | 0.15 | **0.07** | 0.12 | 0.11 | **0.07** | 0.14 | 0.15 | 0.33 | 0.20 | 0.16 | **-0.01** | 0.35 | 0.32 | 0.19 | 0.29 | 0.36 | 0.23 | 0.14 | 0.13 | 0.23 |
| LV-edu | 0.12 | 0.12 | **0.06** | 0.12 | 0.10 | **0.07** | 0.13 | 0.14 | 0.29 | 0.20 | 0.15 | **0.02** | 0.31 | 0.34 | 0.08 | 0.17 | 0.15 | 0.08 | 0.16 | 0.41 | 0.29 |
| LV-ons | 0.16 | 0.16 | 0.11 | 0.19 | 0.18 | 0.20 | 0.12 | 0.14 | 0.34 | 0.24 | 0.20 | **0.01** | 0.30 | 0.38 | 0.08 | 0.15 | 0.09 | **0.06** | 0.14 | 0.13 | 0.22 |
| LV-all | 0.18 | 0.17 | 0.09 | 0.16 | 0.16 | 0.17 | 0.13 | 0.12 | 0.35 | 0.21 | 0.19 | **0.00** | 0.38 | 0.41 | 0.17 | 0.29 | 0.35 | 0.21 | 0.13 | 0.41 | 0.26 |
| LV-PCA | 0.24 | 0.23 | 0.20 | 0.27 | 0.25 | 0.25 | 0.44 | 0.30 | 0.44 | 0.44 | 0.37 | 0.09 | 0.39 | 0.46 | 0.18 | 0.18 | 0.12 | 0.08 | 0.21 | 0.20 | 0.32 |
| LV-PCA-age | 0.29 | 0.27 | 0.20 | 0.25 | 0.26 | 0.24 | 0.44 | 0.29 | 0.48 | 0.43 | 0.38 | 0.08 | 0.47 | 0.47 | 0.32 | 0.35 | 0.36 | 0.24 | 0.20 | 0.19 | 0.32 |
| LV-PCA-edu | 0.23 | 0.22 | 0.19 | 0.26 | 0.24 | 0.23 | 0.43 | 0.29 | 0.43 | 0.43 | 0.37 | 0.11 | 0.41 | 0.49 | 0.17 | 0.23 | 0.16 | 0.09 | 0.22 | 0.43 | 0.39 |
| LV-PCA-ons | 0.24 | 0.23 | 0.20 | 0.28 | 0.28 | 0.29 | 0.43 | 0.31 | 0.45 | 0.43 | 0.37 | 0.08 | 0.39 | 0.48 | 0.16 | 0.16 | 0.10 | 0.09 | 0.19 | 0.19 | 0.31 |
| LV-PCA-all | 0.27 | 0.25 | 0.19 | 0.27 | 0.26 | 0.27 | 0.42 | 0.28 | 0.47 | 0.42 | 0.38 | 0.08 | 0.47 | 0.52 | 0.29 | 0.35 | 0.35 | 0.24 | 0.19 | 0.43 | 0.36 |

Table A2. Predictive power (predictive R^2^) for each behavioural test using various models. The lesion volume only (LV) and lesion volume plus principal component defined regions (LV-PCA) are shown alone and along with various demographic information. Bold text highlight models that were not significant above chance (p>0.05). Abbreviations: years in education (edu), months post onset (onset), Minimal pairs non-word (PALPA 1), Minimal pairs word (PALPA 2), non-word immediate repetition (PALPA 8 I), non-word delayed repetition (PALPA 8 D), word immediate repetition (PALPA 9 I), word delayed repetition (PALPA 8 D), Cambridge naming test (CNT), Boston naming test (BNT), forward digit span (Digit F), backward digit span (Digit B), spoken sentence comprehension from comprehensive aphasia test (CAT spoken), spoken word-picture matching (sWPM), written word-picture matching (wWPM), type/token ratio (TTR), camel and cactus picture form (CCTp), 96-synonym judgement task (Synon), words-per-minute (WPM), speech tokens (TOK) and mean length of utterances (MLU).

| Holdout Correlation | PALPA 8 I | PALPA 8 D | PALPA 9 I | PALPA 9 D | CNT | BNT | Digit F | Digit B | CAT-spoken | sWPM | wWPM | TRR | CCTp | Synon | PALPA 1 | PALPA 2 | Ravens | Brix-ton | WPM | TOK | MLU |
| --- | --- | --- | --- | --- | --- | --- | --- | --- | --- | --- | --- | --- | --- | --- | --- | --- | --- | --- | --- | --- | --- |
| LV | **0.21** | 0.35 | **0.18** | **0.20** | 0.32 | 0.28 | 0.33 | 0.38 | 0.52 | 0.34 | 0.28 | **0.11** | 0.52 | 0.56 | 0.30 | 0.37 | 0.30 | **0.24** | 0.31 | 0.30 | 0.30 |
| LV-age | 0.34 | 0.38 | 0.26 | **0.22** | 0.31 | 0.45 | 0.36 | 0.37 | 0.52 | 0.34 | 0.34 | **0.16** | 0.59 | 0.56 | 0.43 | 0.54 | 0.53 | 0.47 | 0.32 | 0.30 | 0.33 |
| LV-edu | **0.23** | 0.33 | **0.19** | **0.22** | 0.29 | **0.25** | 0.32 | 0.37 | 0.51 | 0.34 | 0.29 | **0.10** | 0.55 | 0.58 | 0.26 | 0.40 | 0.36 | 0.26 | 0.31 | 0.60 | 0.38 |
| LV-ons | **0.00** | 0.28 | **0.07** | **0.08** | 0.35 | 0.34 | **0.25** | 0.36 | 0.55 | 0.48 | 0.26 | **0.13** | 0.50 | 0.61 | 0.27 | 0.37 | 0.28 | **0.23** | 0.35 | 0.30 | 0.30 |
| LV-all | **0.18** | 0.34 | **0.03** | **0.11** | 0.39 | 0.37 | **0.15** | 0.33 | 0.56 | 0.45 | 0.33 | **0.11** | 0.56 | 0.63 | 0.40 | 0.52 | 0.52 | 0.45 | 0.35 | 0.60 | 0.39 |
| LV-PCA | **0.23** | 0.47 | **0.21** | 0.38 | 0.39 | 0.40 | 0.66 | 0.54 | 0.66 | 0.65 | 0.31 | 0.28 | 0.43 | 0.64 | **0.18** | 0.41 | 0.33 | 0.27 | 0.36 | 0.36 | 0.40 |
| LV-PCA-age | 0.41 | 0.51 | 0.29 | 0.41 | 0.50 | 0.48 | 0.60 | 0.53 | 0.69 | 0.65 | 0.38 | 0.27 | 0.54 | 0.68 | 0.49 | 0.59 | 0.56 | 0.47 | 0.36 | 0.32 | 0.44 |
| LV-PCA-edu | 0.27 | 0.45 | **0.24** | 0.42 | 0.48 | 0.47 | 0.65 | 0.53 | 0.65 | 0.65 | 0.32 | 0.32 | 0.43 | 0.66 | **0.20** | 0.44 | 0.39 | 0.28 | 0.34 | 0.60 | 0.45 |
| LV-PCA-ons | **0.13** | 0.47 | **0.10** | 0.30 | 0.46 | 0.45 | 0.64 | 0.54 | 0.66 | 0.65 | 0.29 | 0.27 | 0.42 | 0.59 | **0.18** | 0.39 | 0.30 | 0.28 | 0.36 | 0.33 | 0.39 |
| LV-PCA-all | 0.33 | 0.49 | **0.20** | 0.35 | 0.50 | 0.50 | 0.60 | 0.52 | 0.68 | 0.64 | 0.35 | **0.24** | 0.51 | 0.63 | 0.49 | 0.57 | 0.55 | 0.48 | 0.37 | 0.58 | 0.46 |
